# ANXA2 promotes NLRP3 inflammasome activation and neuronal pyroptosis after intracerebral hemorrhage

**DOI:** 10.3389/fimmu.2026.1759365

**Published:** 2026-05-26

**Authors:** Qingyuan Wu, Yuetao Wen, Yiqing Shen, XiangYu Chen, WenSong Yang, Limin Ma, Peng Xie

**Affiliations:** 1 National Health Commission NHC Key Laboratory of Diagnosis and Treatment on Brain Functional Diseases, The First Affiliated Hospital of Chongqing Medical University, Chongqing, China; 2 Department of Neurology, Chongqing University Three Gorges Hospital, Chongqing, China; 3 Department of Neurosurgery, Chongqing University Jiangjin Hospital, Chongqing, China; 4 Department of Geriatrics, Chongqing University Three Gorges Hospital, Chongqing, China

**Keywords:** AnxA2, intracerebral hemorrhage, neuroinflammation, NLRP3 inflammasome, pyroptosis

## Abstract

**Background:**

Intracerebral hemorrhage (ICH) is a severe form of stroke lacking effective pharmacotherapy, in part because upstream regulators initiating secondary brain injury are not well understood. Pyroptosis mediated by activation of the NLRP3 inflammasome is a major contributor to neuronal death after ICH. However, the upstream mechanisms remain to be fully elucidated.

**Methods:**

We performed integrative transcriptomic–proteomic profiling of mouse ICH brain tissues with *in vivo* functional validation. Annexin A2 (ANXA2), identified as a hub protein, was silenced via genetic knockdown. Neurological function, brain pathology, and pyroptotic signaling were assessed by behavioral tests, histology, Western blotting, immunofluorescence, and co-immunoprecipitation.

**Results:**

Multi-omics and network analyses identified ANXA2 as a prominently upregulated hub protein after ICH. Co-immunoprecipitation demonstrated an association between ANXA2 and NLRP3, while ANXA2 silencing reduced NLRP3 inflammasome activation, decreased GSDMD cleavage and IL-1β/IL-18 secretion and significantly improved neurological function while alleviating brain injury.

**Conclusions:**

This study reveals a previously unrecognized ANXA2–NLRP3–pyroptosis pathway in ICH, revealing a neuronal–immune convergence mechanism in inflammasome regulation. These findings provide new insight into neuronal pyroptosis after ICH and underscore ANXA2 as a predominantly neuronal factor associated with inflammasome activation in hemorrhagic stroke.

## 1 Background

ICH is a catastrophic form of stroke caused by the rupture of cerebral vessels, resulting in hemorrhage within the brain tissue. It accounts for approximately 15%–20% of all strokes worldwide and is associated with high mortality and severe long-term neurological deficits (1, 2). Despite substantial advances in surgical evacuation and neurocritical care, no pharmacological strategy has yet achieved proven clinical efficacy, a gap that stems largely from an incomplete delineation of the secondary injury cascades initiated by the primary hemorrhagic insult (3).

Secondary brain injury after ICH is driven by a multifaceted network of pathological processes, including neuroinflammation, blood–brain barrier breakdown, oxidative stress, and several forms of regulated cell death (4). Among these, pyroptosis, an inflammasome-dependent and highly pro-inflammatory mode of programmed cell death, s increasingly recognized as a pivotal driver of neuronal degeneration and perihematomal edema (5, 6). In this pathway, danger-associated molecular patterns of cytosolic inflammasomes containing pattern recognition receptors such as NLRP1 (7), NLRP3 (8), or AIM2 (9), which recruit the adaptor protein ASC to enable caspase-1 activation. Activated caspase-1 subsequently cuts gasdermin D (GSDMD), liberating its N-terminal fragment to generate transmembrane pores, which lead to cell swelling, osmotic rupture, and the secretion of IL-1β and IL-18 into the extracellular space. These events propagate inflammatory signaling and exacerbate neuronal injury within the perihematomal region (10, 11).

Building on this cascade, the NLRP3 inflammasome has emerged as the predominant inflammasome complex driving neuronal pyroptosis (12). Although NLRP3 inflammasome activation is recognized as a critical driver of post-ICH pathology (13), its upstream regulatory mechanisms within neurons remain poorly defined. Emerging evidence suggests that targeting these upstream checkpoints may offer novel therapeutic opportunities to limit pyroptosis and attenuate neuroinflammation (13, 14).

Annexin A2 (ANXA2), a calcium-dependent phospholipid-binding protein involved in membrane trafficking, cytoskeletal regulation, and immune modulation, has been implicated in inflammasome regulation across various neurological contexts (15, 16). However, the precise function of ANXA2 in ICH pathogenesis, and its potential regulation of secondary brain injury after ICH via NLRP3 inflammasome–dependent pyroptosis, is still unclear.

In this work, we integrated transcriptomic and proteomic analyses of brain tissues from a murine ICH model to identify key molecular regulators of secondary brain injury. We subsequently evaluated the functional role of ANXA2, which emerged as a critical hub protein, in ICH progression through genetic knockdown approaches and neurological assessments. Finally, we investigated the mechanistic link between ANXA2 and neuronal pyroptosis, with a focus on its direct interaction with NLRP3 inflammasome signaling.

## 2 Materials and methods

### 2.1 Animals

Male C57BL/6 mice (7–8 weeks old, 18–22 g body weight) were purchased from Ensiweier (Chengdu, China) and kept under pathogen-free conditions at the Laboratory Animal Center of Chongqing Medical University. The experimental procedures were ethically approved by the Animal Ethics Committee of Chongqing Medical University and strictly adhered to the National Institutes of Health standards for laboratory animal care and use. Animals were maintained under controlled environmental conditions (temperature: 22 ± 2 °C, humidity: 50-60%) with a standard 12hour light/dark cycle, providing unrestricted access to laboratory diet and drinking water.

### 2.2 Induction of ICH

ICH was induced by stereotaxic injection of type IV-S clostridial collagenase (0.075 U in sterile saline; Sigma-Aldrich, USA) into the right striatum as previously described (17). Mice received sodium pentobarbital anesthesia (50 mg/kg, intraperitoneally) before being secured in stereotaxic equipment. A burr hole was created at coordinates 0.6 mm anterior, 2.3 mm lateral, and 3.7 mm ventral from bregma. Collagenase was administered slowly over 5 min period using a Hamilton syringe, with the needle left in place for an additional 5 min to minimize backflow. Sham-operated controls were injected with the same volume of sterile saline.

### 2.3 Transcriptomic analysis

Perihematomal brain samples were harvested from five ICH mice and five sham controls. Total RNA was extracted with TRIzol reagent (Invitrogen, USA), and its concentration and purity were measured using a NanoDrop spectrophotometer (Thermo Fisher Scientific, USA). cDNA libraries were prepared with the NEBNext^®^ Ultra™ RNA Library Prep Kit for Illumina^®^ (New England Biolabs, USA) and sequenced in paired-end mode on the Illumina NovaSeq 6000 platform (Illumina, USA). After quality filtering of raw reads, the cleaned sequences were mapped to the mouse reference genome (GRCm38) using HISAT2. Differential expression analysis was performed using DESeq2. Genes with |log_2_(fold change)| > 0.45 and adjusted p < 0.05 were considered differentially expressed genes (DEGs).

### 2.4 Proteomic analysis

Perihematomal brain tissue from three ICH and three sham mice was homogenized in lysis buffer (100 mM NH_4_HCO_3_, 8 M urea, 0.2% SDS). The homogenates underwent sonication and centrifugation (12,000 × g, 15 min, 4 °C). Supernatants were treated with 10 mM dithiothreitol (DTT), alkylated using iodoacetamide, and precipitated with prechilled acetone. The protein pellets were washed with chilled acetone, reconstituted in 0.1 M triethylammonium bicarbonate supplemented with 6 M urea, enzymatically digested using trypsin, and labeled with Tandem Mass Tags (TMT; Thermo Fisher Scientific, USA). Peptide analysis was performed via LC-MS/MS using an Orbitrap mass spectrometer (Novogene, China). Spectral data were searched against the UniProt Mus musculus reference database for protein identification and quantification. Proteins with |log_2_(fold change)| > 0.5 and adjusted p < 0.05 were considered differentially expressed proteins (DEPs).

### 2.5 Protein-protein interaction network and hub gene analysis

Combined DEGs and DEPs underwent PPI network construction through the STRING database (v11.5) applying a confidence threshold of ≥0.4. Network visualization was achieved using Cytoscape software (v3.10.0), while hub gene identification was conducted via the CytoHubba plugin according to degree centrality metrics. Functional enrichment analyses were conducted through DAVID and Metascape platforms.

### 2.6 Quantitative real-time PCR

Total RNA from perihematomal brain regions was converted to cDNA using the PrimeScript™ RT reagent kit (Takara, Japan). Quantitative PCR was conducted with SYBR^®^ Green Master Mix (Takara, Japan) on a QuantStudio™ 5 Real-Time PCR System (Applied Biosystems, USA) (18). GAPDH served as the internal control, and relative gene expression was calculated via the 2^−ΔΔCt method. The ANXA2 primer sequences were: forward 5′-ATGTCCCAAGTGGATCAGC-3′, reverse 5′-ACAGGGCTTGTCTGAATG-3′.

### 2.7 Western blotting and co-immunoprecipitation

Protein samples (30–50 µg) from perihematomal brain tissue were resolved by SDS-PAGE and transferred to PVDF membranes (Millipore, USA). Following blocking with 5% skim milk, membranes underwent overnight incubation at 4 °C with primary antibodies targeting: ANXA2 (rabbit, 11256-1-AP, Proteintech, 1:3000), GSDMD (mouse, sc-393656, Santa Cruz, 1:1000), NLRP3 (rabbit, 30109-1-AP, Proteintech, 1:1000), ASC (rabbit, 340097, ZenBio, 1:1000), Caspase-1/Cleaved Caspase-1 (rabbit, WL03450, Wanleibi, 1:1000), IL-1β (rabbit, 516288, ZenBio, 1:1000), IL-18 (rabbit, 10663-1-AP, Proteintech, 1:1000), and GAPDH (rabbit, 10494-1-AP, Proteintech, 1:3000). After incubation with HRP-conjugated secondary antibodies, immunoreactive bands were detected using enhanced chemiluminescence (Bio-Rad, USA) and visualized with a Fusion imaging system (Vilber Lourmat, France) (19).

For Co-IP, perihematomal brain tissues were processed as previously described (20). In brief, tissue samples were mechanically disrupted in RIPA extraction buffer supplemented with protease and phosphatase inhibitors, maintained on ice, and subjected to centrifugation at 12,000 × g for 15 minutes at 4 °C. The resulting supernatants were precleared using control IgG and Protein A/G magnetic beads (MedChemExpress, China), followed by overnight incubation at 4 °C with either anti-ANXA2 or anti-NLRP3 primary antibodies. Subsequently, immunoprecipitated complexes were isolated via Protein A/G bead capture, eluted, and subjected to Western blot analysis.

### 2.8 Immunofluorescence staining

Frozen brain sections (15 μm) from 4% paraformaldehyde-fixed, sucrose-cryoprotected tissues were processed for immunofluorescence as previously described (20). Multiplexed immunostaining employed tyramide signal amplification via the BeyoTSA™ system (488/555/647/DAPI, Beyotime, China) per manufacturer guidelines. Following antigen unmasking and blocking procedures, three consecutive labeling rounds were executed, comprising overnight primary antibody exposure, HRP-linked secondary antibody treatment, and tyramide fluorophore development (TSA-647, TSA-555, or TSA-488). Between cycles, bound antibodies were stripped using the buffers provided in the kit.

Fluorescent images were acquired on a Nikon A1R confocal microscope (Nikon, Japan) with sequential scanning. Co-localization was assessed qualitatively based on signal overlap. Quantitative analysis was performed using ImageJ (NIH, USA) to measure fluorescence intensity within regions of interest defined by consistent anatomical landmarks.

### 2.9 Lentiviral-mediated ANXA2 knockdown

Lentiviral vectors encoding shRNA targeting mouse ANXA2 (target sequence: 5′-GTATGATGCTTCGGAACTAAA-3′) or scrambled control (5′-TTCTCCGAACGTGTCACGT-3′) were obtained from GK Biotechnology (Wuhan, China). Both vectors co-expressed GFP for infection tracking and had viral titers of 5×10^8^ TU/mL. Using aseptic technique, 2.5 μL of viral suspension was stereotactically delivered to the right striatal region (coordinates: 0.6 mm rostral, 2.3 mm lateral, 3.7 mm deep relative to bregma) via slow infusion (0.25 μL/min) through a 5 μL Hamilton microsyringe. Post-injection, the cannula remained positioned for 5 minutes to prevent backflow. Lentiviral administration was performed 14 days before ICH induction to ensure effective ANXA2 knockdown.

### 2.10 Neurological function assessment

Neurological function was assessed 3 days after ICH establishment utilizing the modified Garcia scale and forelimb positioning assay. The modified Garcia scale evaluates spontaneous activity, limb symmetry, forelimb strength, climbing ability, and responses to tactile and visual stimuli, with scores ranging from 0 to 18, where lower scores indicate more severe neurological deficits (21, 22). The forelimb placement assay measures sensorimotor integration by stimulating the vibrissae and recording correct forelimb placement responses over 10 trials for each side. All behavioral testing was performed by two independent investigators blinded to experimental groups (22).

### 2.11 Statistical analysis

Results are presented as mean ± standard deviation (SD). Two-group statistical evaluations employed unpaired, two-tailed Student’s t-tests. Multi-group analyses utilized one-way analysis of variance (ANOVA) with subsequent Tukey’s *post hoc* testing. Statistical significance was defined as p < 0.05. Data analysis was conducted using IBM SPSS Statistics version 22.0 and GraphPad Prism 9.0 software packages.

## 3 Results

### 3.1 Overview of omics data acquisition and quality assessment

**Figure 1:**
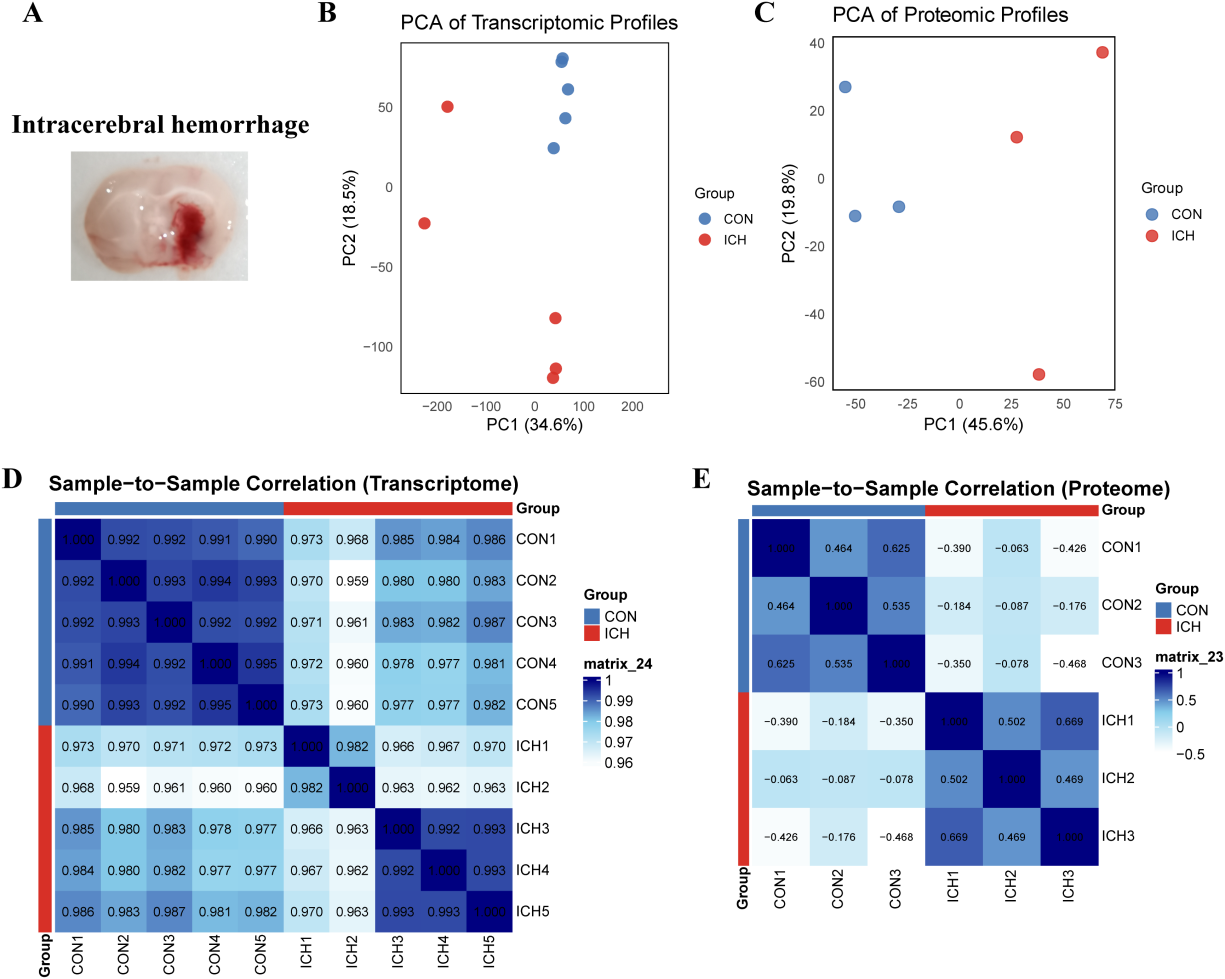
Integrated transcriptomic and proteomic profiling of brain tissue following ICH. **(A)** Representative mouse brain image at 3 days after collagenase-induced ICH, showing a localized hematoma in the striatum. **(B)** PCA of transcriptomic profiles from control (CON) and ICH groups, revealing distinct clustering between conditions. **(C)** PCA of proteomic profiles showing clear separation between CON and ICH samples. **(D)** Pearson correlation heatmap of transcriptomic profiles demonstrating strong intra-group correlations and marked inter-group divergence. **(E)** Pearson correlation heatmap of proteomic profiles demonstrating strong within-group concordance yet higher inter-sample variability.

To confirm the successful establishment of the ICH model, histological analysis was conducted on brain sections obtained three days after collagenase administration. Representative coronal sections revealed a well-defined hemorrhagic lesion with hematoma formation in the right striatum of ICH mice, confirming effective induction of focal intracerebral hemorrhage (**Figure 1A**). For integrated molecular profiling, brain tissues from the ipsilateral striatum were systematically collected and subjected to both transcriptomic and proteomic analyses. RNA sequencing was performed on samples from ICH (n = 5) and sham-operated controls (CON, n = 5), while TMT-based quantitative proteomics was conducted on an independent cohort (ICH, n = 3; CON, n = 3). RNA- sequencing detected 54,533 genes across all samples, and LC–MS/MS-based proteomics identified and quantified 6,013 proteins, providing broad molecular coverage. Principal component analysis (PCA) demonstrated clear separation between ICH and CON groups in both transcriptomic (PC1: 34.6%, PC2: 18.5%) and proteomic (PC1: 45.6%, PC2: 19.8%) datasets, with tight clustering within each group (**Figures 1B, C**). Inter-sample correlation analysis further confirmed dataset reproducibility, showing high within-group correlations for transcriptomic profiles (r = 0.962–0.995) and acceptable technical reproducibility for proteomic profiles (r = 0.469–0.669), while both datasets exhibited distinct inter-group divergence (**Figures 1D, E**). All datasets met standard quality control criteria, providing a robust foundation for subsequent differential expression analyses.

### 3.2 Transcriptomic analysis reveals comprehensive gene expression remodeling

**Figure 2:**
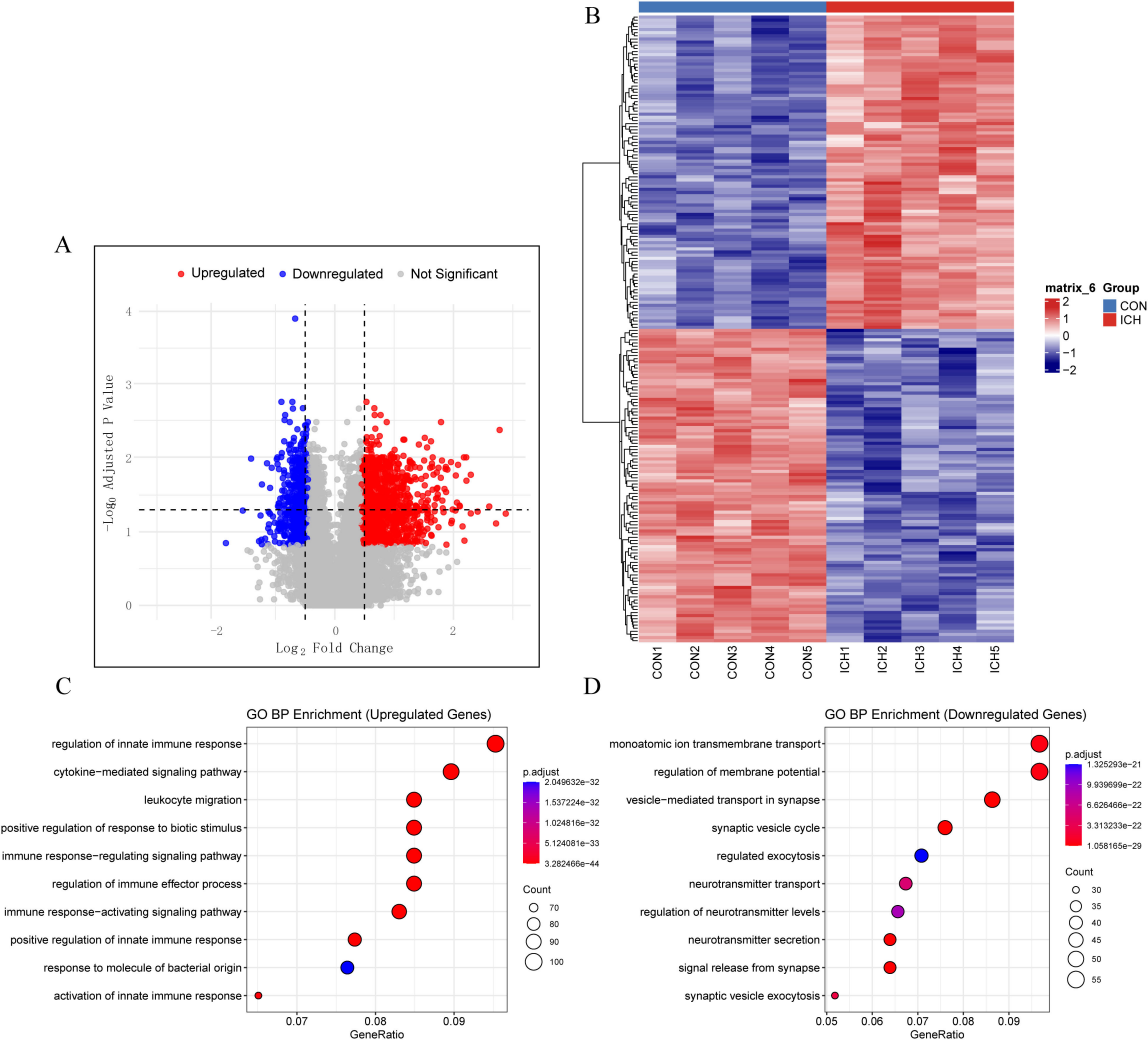
Transcriptomic alterations and functional enrichment analysis following ICH. **(A)** Volcano plot of DEGs between control and ICH groups. Upregulated genes (red), downregulated genes (blue), and non-significant genes (grey) are shown (|log_2_FC| > 0.45, adjusted *P* < 0.01). **(B)** Heatmap of top 50 DEGs illustrating distinct expression patterns and group separation. **(C)** GO biological process enrichment for upregulated DEGs, highlighting innate immune activation, cytokine-mediated signaling, and leukocyte migration. **(D)** GO biological process enrichment for downregulated DEGs, showing suppression of synaptic vesicle cycle, neurotransmitter transport, and membrane potential regulation.

Transcriptomic profiling identified 1,706 DEGs between ICH and control groups under stringent thresholds (|log_2_FC| > 0.45 and adjusted p < 0.05), comprising 1,098 upregulated and 608 downregulated genes. Volcano plot visualization delineated these significantly altered transcripts from the unchanged background, indicating extensive gene expression remodeling (**Figure 2A**). Unsupervised hierarchical clustering revealed distinct expression signatures that clearly segregated ICH from control samples, with well-defined clusters of upregulated (red) and downregulated (blue) genes (**Figure 2B**). Gene Ontology (GO) enrichment analysis revealed a marked polarization of biological processes. Upregulated DEGs were predominantly enriched in neuroinflammation associated pathways (23, 24), including regulation of innate immune response, cytokine-mediated signaling, and leukocyte migration (all adjusted *p* < 0.001) (**Figure 2C**). In contrast, downregulated DEGs were significantly associated with neuronal functions (25) such as ion transmembrane transport, regulation of membrane potential, and synaptic vesicle cycle (all adjusted *p* < 0.001), indicative of impaired neuronal excitability and disrupted synaptic transmission (**Figure 2D**).

### 3.3 Differential proteomic profiling reveals ICH-associated protein signatures

**Figure 3:**
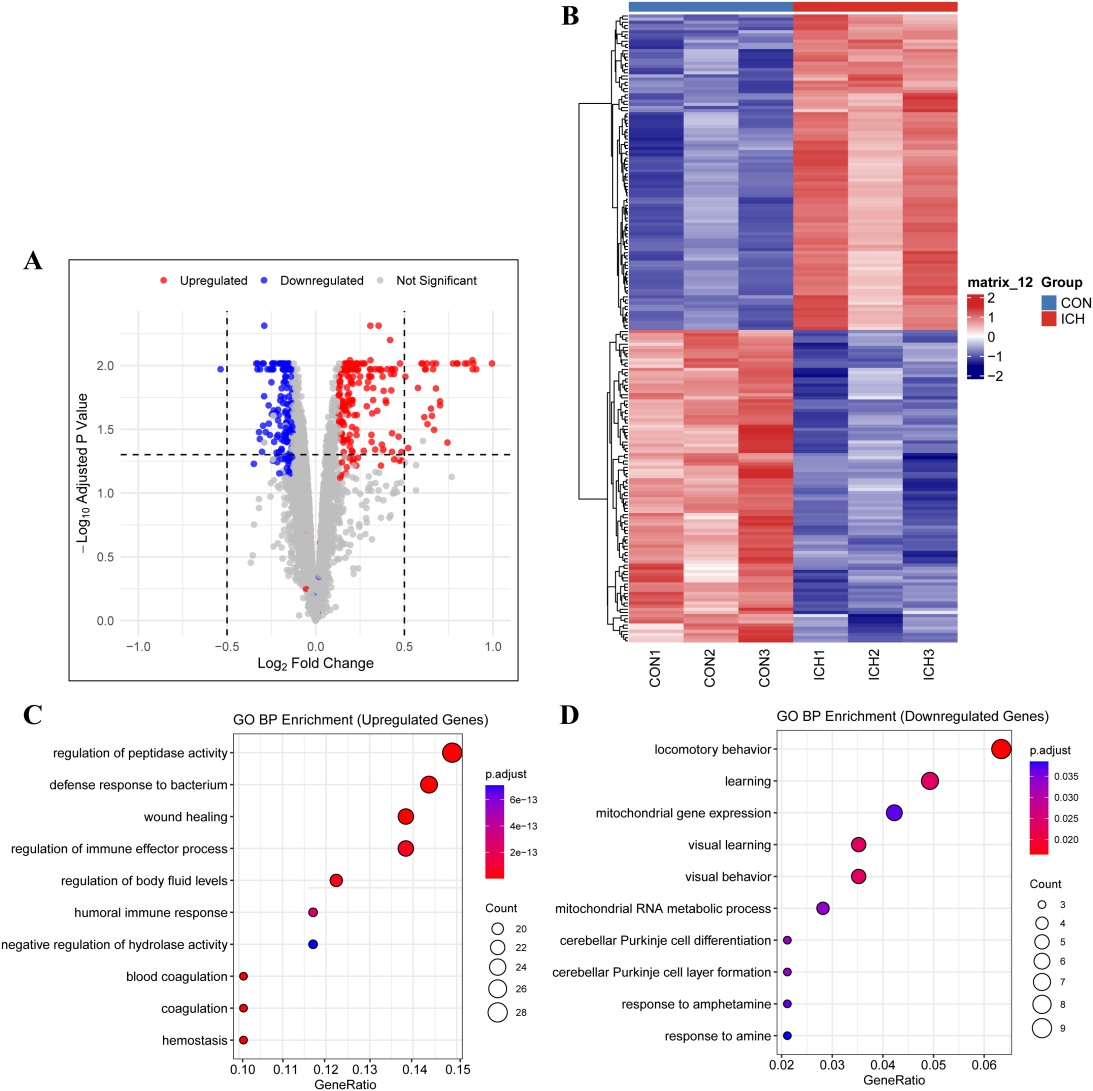
Proteomic alterations and functional enrichment analysis following ICH. **(A)** Volcano plot of DEPs between CON and ICH groups, with significantly upregulated proteins in red, downregulated proteins in blue, and non-significant proteins in grey (|log_2_FC| > 0.5, adjusted *P* < 0.05). **(B)** Heatmap of top 50 DEPs showing distinct proteomic profiles and separation between groups. **(C)** GO biological process enrichment for upregulated DEPs, highlighting immune response regulation, coagulation, and wound healing. **(D)** GO biological process enrichment for downregulated DEPs, indicating reduced activity in learning, memory, and mitochondrial gene expression.

Proteomic profiling yielded 344 DEPs (|log_2_FC| > 0.5 and adjusted *p* < 0.05), with 194 upregulated and 150 downregulated species. Volcano and hierarchical clustering analyses similarly revealed marked separation between ICH and control samples, with coherent clusters of upregulated and downregulated proteins (**Figures 3A, B**). Functional enrichment analysis of DEPs mirrored transcriptomic findings. Upregulated proteins were predominantly enriched in immune and inflammation related processes (26), including regulation of peptidase activity, defense response to bacterium, and wound healing (all adjusted *p* < 0.01) (**Figure 3C**). Conversely, downregulated proteins were significantly enriched in pathways related to locomotor behavior, learning (27), and mitochondrial gene expression (28) (all adjusted *p* < 0.05), reflecting impairments in neurological function and cellular energy metabolism (**Figure 3D**).

Taken together, integrative analysis of transcriptomic and proteomic data revealed a coordinated molecular response to ICH, characterized by pronounced activation of immune and inflammatory programs and concomitant suppression of neuronal signaling and metabolic pathways at both transcript and protein levels.

### 3.4 Integrated multi-omics analysis and hub gene identification

**Figure 4:**
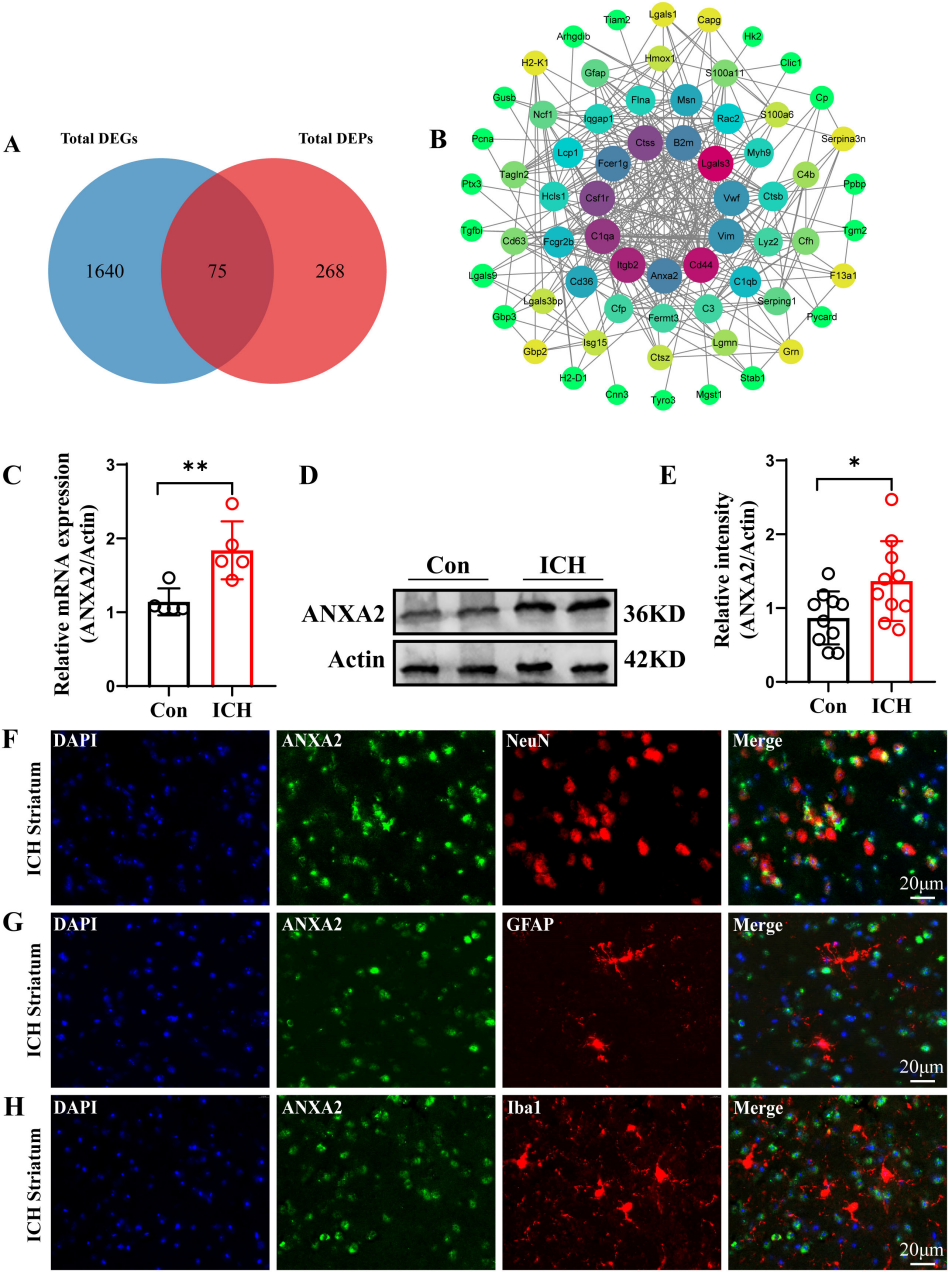
Integrated transcriptomic–proteomic analysis identifies shared differentially expressed molecules and highlights neuronal upregulation of ANXA2 after ICH. **(A)** Venn diagram showing the overlap between DEGs and DEPs in ICH, with 75 molecules consistently altered at both transcript and protein levels. **(B)** PPI network of the 75 overlapping molecules, with hub proteins clustered centrally (purple; ANXA2, CD44, ITGB2, C3, LGALS3), surrounded by other key network components (blue) and peripheral interactors (green). **(C)** qRT-PCR analysis of ANXA2 mRNA levels in control and ICH brain tissues (n = 5 per group; unpaired Student’s t-test; **P = 0.0069). **(D)** Representative Western blot images of ANXA2 and β-actin in control and ICH groups. **(E)** Quantification of ANXA2 protein normalized to β-actin(n = 10 per group; unpaired Student’s t-test; *P = 0.0256). **(F–H)** Representative double immunofluorescence images of ANXA2 (green) with **(F)** NeuN, **(G)** GFAP, and **(H)** Iba1 (red) in the perihematomal region. Co-localization was assessed qualitatively based on signal overlap. Nuclei were counterstained with DAPI (blue). (Scale bar = 20μm. *P < 0.05, **P < 0.01).

To identify robust molecular targets for ICH intervention, we performed integrative analysis of transcriptomic and proteomic datasets. Comparative profiling revealed 1,640 DEGs and 268 DEPs, with 75 molecules consistently dysregulated at both transcriptomic and proteomic levels (**Figure 4A**). These cross-validated molecules represent high-confidence candidates that are reliably altered following ICH across multiple molecular layers.

To delineate the functional relationships among these 75 overlapping molecules, a PPI network was generated via the STRING platform, applying an interaction confidence threshold of >0.4. The resulting network exhibited densely interconnected clusters, indicative of coordinated responses to ICH injury (**Figure 4B**). Hub gene analysis using cytoHubba algorithm identified 11 key proteins based on degree centrality, including Cd44, Anxa2, Itgb2, Ctss, C3, Lgals3, among others, which occupied central positions within the PPI network and exhibited extensive connectivity (**Figure 4B**). These hub proteins are primarily involved in complement activation, immune cell adhesion, and inflammatory signaling pathways.

### 3.5 ANXA2 emerges as a key hub protein with therapeutic potential

Among these hub proteins, ANXA2 emerged as a compelling candidate owing to its high degree centrality within the PPI network and marked upregulation in both omics datasets. It has been implicated in maintaining blood–brain barrier integrity (29), modulating neurovascular inflammation (30), and has also been associated with multiple cerebrovascular disorders (31), processes that were prominently enriched in both our transcriptomic and proteomic analyses of ICH. Nevertheless, the precise function of ANXA2 in ICH has yet to be clarified.

To validate these findings, ANXA2 expression was first examined at the mRNA level by quantitative PCR, which revealed a significant increase in ICH mice compared with controls (**Figure 4C**). Protein level assessment by Western blotting further demonstrated marked upregulation of ANXA2 in ICH brain tissues (**Figures 4D, E**). Immunofluorescence co-localization analysis showed that ANXA2 signals were predominantly observed in NeuN-positive neurons within the perihematomal striatum (**Figure 4F**). In contrast, limited overlap was detected between ANXA2 and GFAP-positive astrocytes (**Figure 4G**) or Iba1-positive microglia (**Figure 4H**). These findings suggest a predominantly neuronal localization pattern of ANXA2 following ICH, supporting its identification as a hub protein in our multi-omics analysis.

### 3.6 ANXA2 knockdown attenuates ICH-induced brain injury and neurological deficits

**Figure 5:**
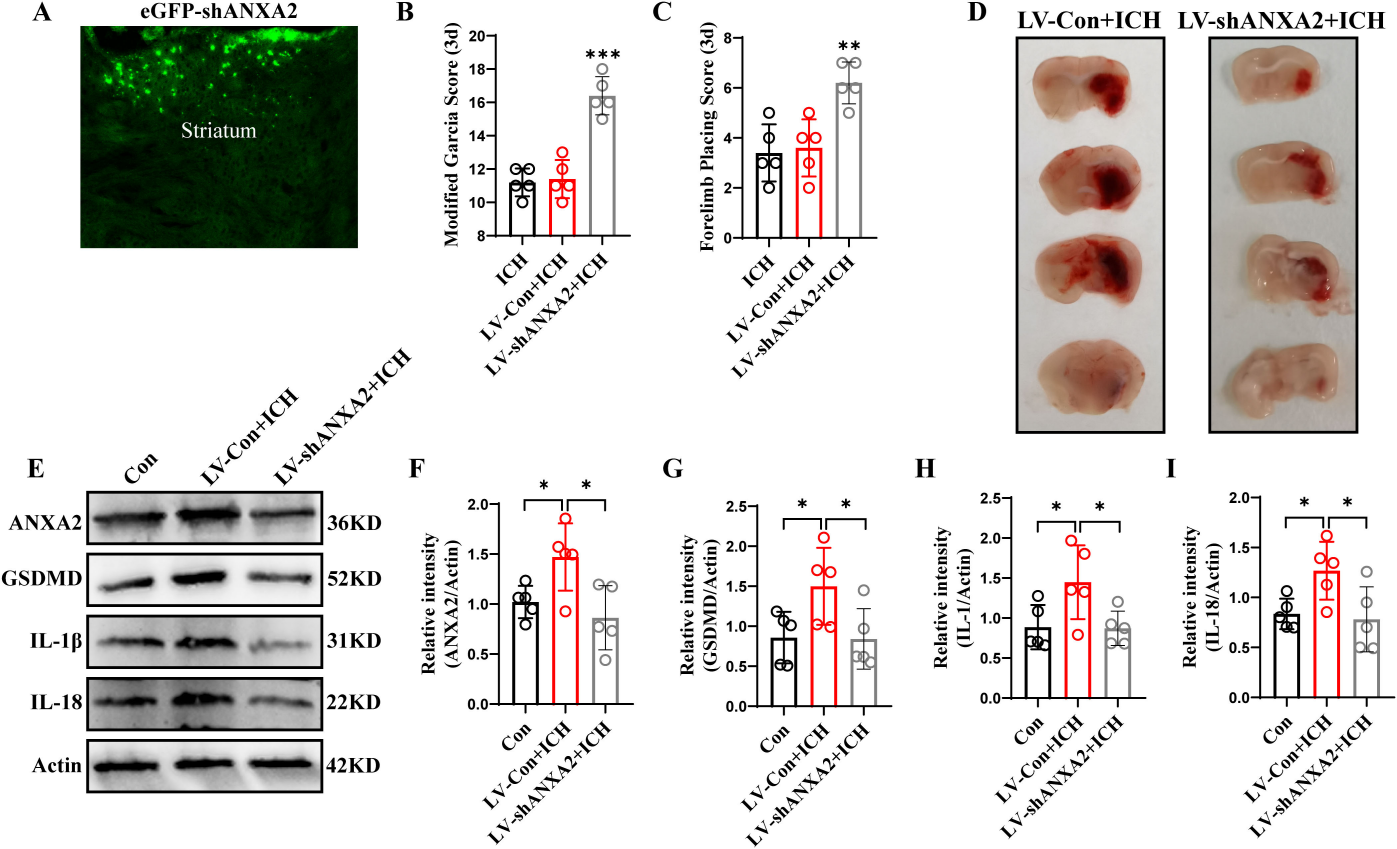
ANXA2 silencing alleviates brain injury by inhibiting pyroptotic pathways in ICH. **(A)** Representative fluorescence image showing eGFP expression in the striatum after LV-shANXA2 injection. **(B, C)** Neurological scores at day 3 post-ICH assessed by the **(B)** modified Garcia test and **(C)** forelimb placing test (n = 5 per group; one-way ANOVA (Tukey’s test); **P = 0.0034, ***P = 0.0001). **(D)** Representative brain sections showing reduced hematoma volume in LV-shANXA2-treated mice compared with LV-Con controls. **(E)** Representative Western blots of ANXA2, GSDMD, IL-1β, and IL-18 in perihematomal brain tissues. **(F–I)** Quantification of protein levels normalized to β-actin for **(F)** ANXA2, **(G)** GSDMD, **(H)** IL-1β, and **(I)** IL-18 (n = 5 per group; one-way ANOVA (Tukey’s test); *P < 0.05, **P < 0.01, ***P < 0.001).

To clarify the functional contribution of ANXA2 to ICH, lentiviral-mediated ANXA2 knockdown (LV-shANXA2) was performed via stereotactic injection prior to ICH induction. Efficient ANXA2 knockdown was confirmed by immunofluorescence staining in the striatum (**Figure 5A**). Neurological assessment 3 days post-ICH revealed that ICH mice exhibited significant neurological impairment as measured by modified Garcia scores. Remarkably, ANXA2 knockdown significantly improved neurological function compared to control ICH mice (**Figure 5B**). Similarly, forelimb placing deficits induced by ICH were substantially ameliorated following ANXA2 silencing (**Figure 5C**). Macroscopic examination of brain tissues demonstrated that ANXA2 knockdown markedly reduced hematoma formation compared to control ICH mice (**Figure 5D**), indicating that ANXA2 contributes to hemorrhagic injury severity. Collectively, these results indicate that ANXA2 knockdown mitigates cerebral damage severity and enhances neurological recovery in ICH animals.

### 3.7 ANXA2 mediates ICH-induced pyroptotic inflammation

Given the prominent inflammatory signatures identified in our multi-omics analysis and emerging evidence implicating ANXA2 in pyroptosis regulation (32, 33), the role of ANXA2 in ICH-related pyroptotic signaling was investigated. Western blotting demonstrated a marked increase in GSDMD expression following ICH, which was markedly attenuated by ANXA2 knockdown (**Figures 5E–G**). Consistent with pyroptosis-associated inflammatory responses, levels of the pivotal pro-inflammatory cytokines IL-1β and IL-18 were markedly increased in ICH mice but were effectively suppressed upon ANXA2 silencing (**Figures 5H, I**). These findings suggest that ANXA2 acts as an essential regulator of pyroptotic inflammation during ICH, thereby providing a mechanistic link between our multi-omics findings and inflammatory brain injury.

### 3.8 ANXA2 regulates pyroptosis through NLRP3 inflammasome signaling

**Figure 6:**
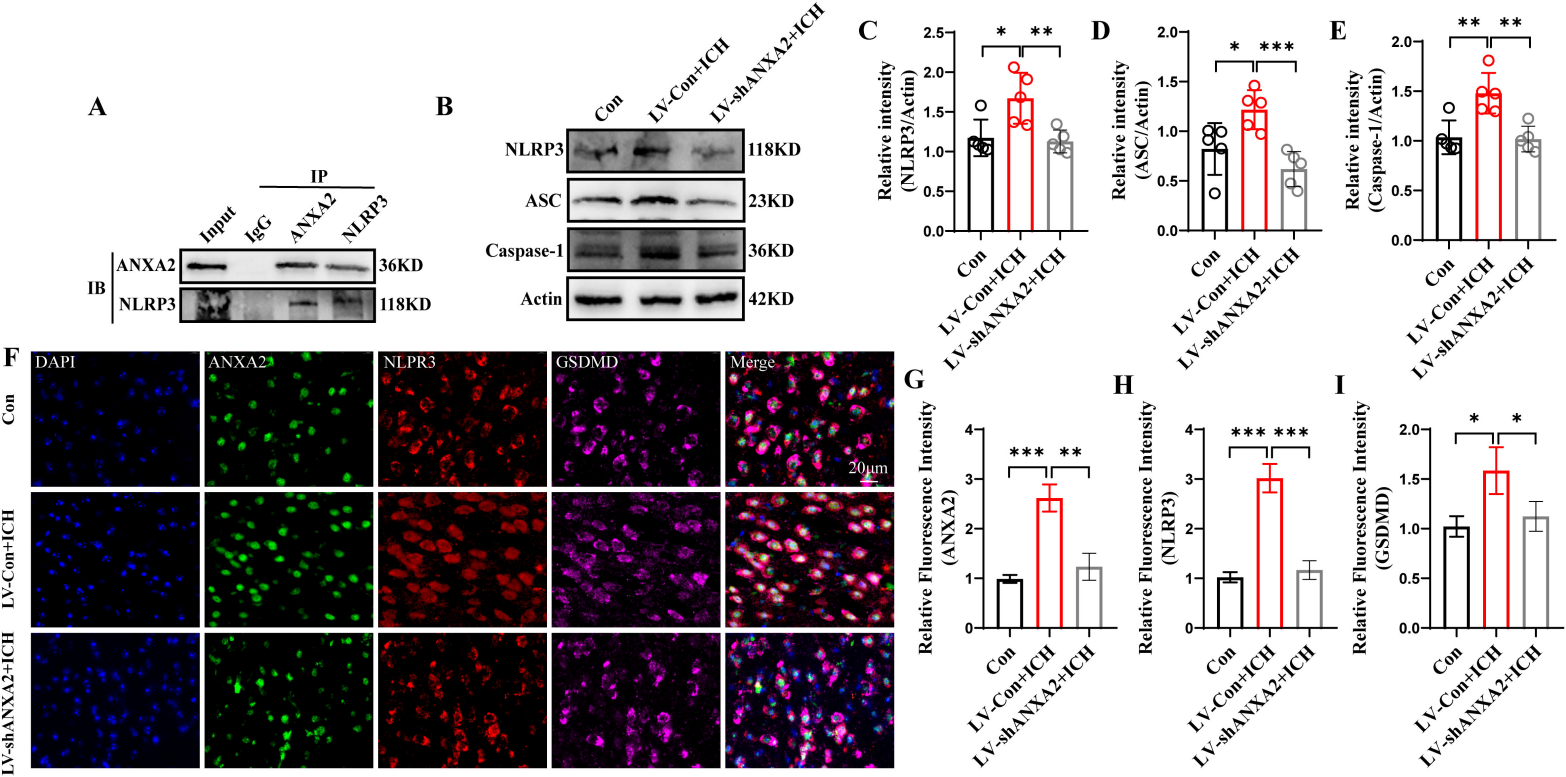
ANXA2 interacts with NLRP3 and regulates inflammasome activation after ICH. **(A)** Co-immunoprecipitation showing physical interaction between ANXA2 and NLRP3 in perihematomal brain tissues. **(B)** Representative Western blots of NLRP3, ASC, and Caspase-1 in control, LV-Con+ICH, and LV-shANXA2+ICH groups. **(C–E)** Quantification of NLRP3 **(C)**, ASC **(D)**, and Caspase-1 **(E)** protein levels normalized to β-actin (n = 5 per group; one-way ANOVA (Tukey’s test); *P < 0.05, **P < 0.01, ***P < 0.001). **(F)** Representative immunofluorescence images showing ANXA2 (green), NLRP3 (red), and GSDMD (magenta) co-localization in the perihematomal region, nuclei counterstained with DAPI (blue). Scale bar = 20μm. **(G–I)** Quantification of fluorescence intensity for ANXA2 **(G)**, NLRP3 **(H)**, and GSDMD **(I)** (n = 5 per group; one-way ANOVA (Tukey’s test); *P < 0.05, **P < 0.01, ****P < 0.0001).

To explore the molecular basis of ANXA2-mediated pyroptosis, the potential association between ANXA2 and canonical inflammasome signaling pathways was investigated. Given that the NLRP3 inflammasome is a key regulator of GSDMD-mediated pyroptosis in neurological conditions, it was prioritized as a candidate pathway. Co-IP analysis showed that ANXA2 was associated with NLRP3 in perihematomal brain tissue (**Figure 6A**), suggesting a potential association between the two proteins. Western blotting further revealed marked upregulation of NLRP3 (**Figures 6B, C**), the ASC adaptor protein (**Figure 6D**), and Caspase-1 (**Figure 6E**) in ICH mice, all of which were significantly reduced following ANXA2 knockdown. Immunofluorescence triple labeling showed that ANXA2, NLRP3, and GSDMD signals were spatially associated in the perihematomal region, with increased fluorescence intensity in the LV-Con + ICH group compared to controls, and substantial attenuation after ANXA2 silencing (**Figure 6F**). Quantitative analyses confirmed these reductions across all three proteins (**Figures 6G–I**).

Together, these data suggest that ANXA2 is associated with NLRP3 inflammasome activation and downstream pyroptotic signaling in ICH, supporting a potential regulatory involvement of ANXA2 in inflammatory brain injury.

## 4 Discussion

Our findings reveal that ANXA2 serves a pivotal role in ICH-associated neuronal death through a previously uncharacterized mechanism involving NLRP3 inflammasome activation and pyroptosis. Although ANXA2 has been implicated in diverse cerebrovascular disorders (20, 29), prior investigations have predominantly focused on its roles in maintaining blood–brain barrier integrity and regulating vascular permeability, leaving the roles of predominantly neuronal ANXA2 in inflammasome activation and pyroptotic injury largely undefined. By integrating high-resolution transcriptomic and proteomic analyses, we identify ANXA2 as a central mediator linking neuroinflammatory signaling with GSDMD-dependent pyroptotic cell death, thereby redefining its pathological role beyond membrane association and suggesting new therapeutic avenues for hemorrhagic stroke.

Pyroptosis is a major driver of secondary injury in cerebrovascular disorders. Our multi-omics analysis revealed significant enrichment of pyroptosis related genes and inflammatory pathways after ICH, including innate immune activation, cytokine signaling, and inflammasome assembly. PPI network analysis identified 11 hub proteins, among which CD44 (34), ITGB2 (35), C3 (36), and LGALS3 (37) are functionally linked to NLRP3 inflammasome–mediated pyroptosis. These molecules likely act through complementary mechanisms, linking complement activation (C3), immune cell infiltration (ITGB2), lysosomal–inflammasome signaling (CTSS), and microglial activation (CD44, LGALS3), ultimately converging on GSDMD-mediated membrane pore formation. Within this network, ANXA2 emerges as the missing link connecting membrane dynamics to neuronal inflammasome activation in ICH.

In this study, we observed that ANXA2 expression was substantially elevated in NeuN-expressing neurons throughout the perihematomal area, indicating a predominant neuronal localization. Silencing ANXA2 significantly improved post-ICH neurological function, reduced brain tissue injury, and decreased hematoma volume, underscoring its pathogenic role in ICH progression. Mechanistically, ANXA2 knockdown inhibited GSDMD cleavage and reduced IL-1β and IL-18 release, indicating that ANXA2 modulates neuronal pyroptosis through regulation of downstream inflammasome signaling.

The NLRP3 inflammasome, composed of the NLRP3, ASC, and Caspase-1, initiates pyroptosis in response to pathological stimuli such as extracellular ATP, reactive oxygen species, and potassium efflux (38). Activated caspase-1 cleaves GSDMD to form membrane pores and processes IL-1β and IL-18 into their mature forms (38, 39). Aberrant NLRP3 activation contributes to neuroinflammation and neuronal injury in multiple neurological disorders, including ICH (40), yet its upstream regulatory mechanisms remain incompletely understood.

Our data identify ANXA2 as a predominantly neuronal regulator associated with NLRP3 inflammasome activation. Co-immunoprecipitation demonstrated an association between ANXA2 and NLRP3, and ANXA2 silencing reduced NLRP3, ASC, and Caspase-1 expression, while suppressing GSDMD cleavage and pro-inflammatory cytokine release. Immunofluorescence showed spatial overlap of ANXA2, NLRP3, and GSDMD signals. Together with prior ANXA2–NeuN staining, this suggests a predominant neuronal localization pattern. This co-localization was markedly diminished following ANXA2 silencing. Collectively, these findings support the existence of a previously unrecognized ANXA2–NLRP3–GSDMD signaling axis associated with neuronal inflammasome activation and immune-mediated injury in ICH.

Several limitations should be acknowledged. While the collagenase-induced ICH model is well-established and reproducible, it cannot fully recapitulate the complexity of human disease, highlighting the need for validation in patient-derived samples. In addition, the proteomic analysis was based on a relatively small cohort, which may limit the detection of low-abundance proteins, although transcriptomic and proteomic data showed consistent convergence at the pathway level. Moreover, this study focused on the acute phase (day 3 post-ICH) and did not assess earlier molecular events or long-term pathological consequences. Therefore, the effects of ANXA2 modulation on long-term functional recovery remain to be determined. Finally, ANXA2 modulation was performed prior to ICH induction, and thus the therapeutic potential of post-injury intervention requires further investigation.

## 5 Conclusion

This study identifies an ANXA2–NLRP3–GSDMD signaling axis associated with neuronal pyroptosis in secondary brain injury after ICH. We demonstrate that ANXA2 upregulation is associated with NLRP3 inflammasome activation, while ANXA2 inhibition attenuates brain injury and improves neurological outcomes. These findings provide new insight into the regulation of pyroptosis in ICH.

## Data Availability

The raw data supporting the conclusions of this article will be made available by the authors, without undue reservation.
